# Consumption

**Published:** 1859-12

**Authors:** 


### CONSUMPTION.

More persons died of consumption in New York City during 1857, than in 1856. But judging from newspaper advertisements, we would suppose there was not the slightest necessity for a single individual thus to perish, who could raise a few three cent postage stamps, as two of them will purchase a "*recipe*" for the mastery of this terrible disease. The late and highly vaunted "*medicated inhalation*," that is, the drawing of the fumes of medicines into the lungs, and thus applying them to the very seat of the disease, although "*it appears very reasonable*," (to those who know nothing at all on the subject,) is utterly inefficient, having in two years almost passed from public remembrance.

What are we to think of the value of our medical schools, when the names of hundreds of young physicians were paraded in print as inquiring into the merits of the new practice? It showed the mortifying fact, that multitudes of young men graduate, who are ignorant of the very first principles of the noble and humane art, the name of which they live but to disgrace.

And further, it ought to read a lesson to editors not to lend their aid in building up a system of medicine, of the nature of which they are wholly ignorant. For it cannot be forgotten that almost every secular paper in New York, threw the weight of its influence, by commendatory editorials, towards inducing their readers to place themselves under the treatment; some of these editors receiving as much as three hundred and fifty dollars for a single advertisement, the "editorial notice" included. We do not say that these editors propagated a deliberate falsehood; very likely they believed with the multitude that there was some truth in the "new practice," and that it appeared to them just as "*reasonable*" as it did to the most uneducated John Smith, but a true morality holds them responsible to their readers for commending to them, ignorantly, a worthless remedy, just as responsible, only more seriously so, as for recommending an investment in an unsound company.

All ought to have known that if it had been an efficient remedy, educated physicians of all nations would have hailed it as a boon to humanity, and held up the discoverer as another *Harvey* or *Jenner*, instead of which, a silent contempt has been the universal award.

We repeat here our oft expressed opinion, that no remedy known to man has any radical, permanent efficiency over consumption, except so far as it promotes a healthy digestion of food; whatever does that, makes the first step towards the arrest and cure of the disease. But another step must be taken, or this first one will inevitably fall short of a desired result, and that second step is, *large and moderate out-door activities.* We cannot express the earnestness we feel in having these two ideas taken hold of, not only by the people, but by the medical profession of all lands.

